# Case Report: A missed diagnosis of cardiac amyloidosis using echocardiography due to immunoglobulin light chain amyloidosis with normal wall thickness in the early stage

**DOI:** 10.3389/fcvm.2024.1331157

**Published:** 2024-12-20

**Authors:** Xiaohui Li, Tongge Mu, Yangxue Deng, Yu Zhang, Yun Ti, Lei Zhang

**Affiliations:** ^1^ National Key Laboratory for Innovation and Transformation of Luobing Theory, Jinan, China; ^2^ The Key Laboratory of Cardiovascular Remodeling and Function Research, Chinese Ministry of Education, Chinese National Health Commission and Chinese Academy of Medical Sciences, Jinan, China; ^3^ Department of Cardiology, Qilu Hospital of Shandong University, Jinan, China; ^4^ The First Clinical Medical College of Shandong University of Traditional Chinese Medicine, Jinan, China; ^5^ Department of Cardiology, Yangkuang New Journey General Hospital, Jining, China

**Keywords:** case report, cardiac amyloidosis, light chain, echocardiography, wall thickness

## Abstract

**Background:**

Cardiac amyloidosis (CA) is a challenging diagnosis, particularly when the classic signs, such as increased wall thickness in a non-dilated left ventricle (LV), are absent. This makes the diagnosis more difficult in patients with normal LV wall thickness. We present a case of CA without increased wall thickness and without the characteristic granular sparkling echotexture in a non-dilated LV.

**Case summary:**

A 50-year-old female patient presented with worsening breathlessness on exertion, paroxysmal nocturnal dyspnea, oliguria, and lower-extremity edema. Electrocardiography showed low voltage in the limb leads and a pseudoinfarction pattern in the anterior leads. The echocardiographic evaluation revealed a non-dilated LV with normal wall thickness, no granular sparkling echotexture of the myocardium, a mildly dilated left atrium, restrictive filling (grade 3 diastolic dysfunction), and pericardial effusion. A follow-up quantitative echocardiographic study 2 weeks later showed a slight increase in LV wall thickness (still within the normal range), decreased global longitudinal strain, and a relative “apical sparing” pattern of longitudinal strain in the apex of the LV. After 1 month, LV wall thickness increased beyond the normal range, and the granular sparkling echotexture became evident. Cardiac amyloidosis was subsequently confirmed by delayed gadolinium enhancement on cardiac magnetic resonance imaging, abnormal serum-free light chain levels, positive serum immunofixation, and an extracardiac biopsy positive for amyloid.

**Discussion:**

Patients presenting with normal wall thickness in a non-dilated LV might only be in an early stage of CA. Thus, the diagnosis can be easily overlooked. For smaller individuals, relative wall thickness (RWT) may be a more sensitive indicator for further investigation. In patients presenting with increased RWT, restrictive filling, and pericardial effusion in the absence of other plausible causes, CA should be considered, even in the absence of the classic echocardiographic signs of amyloid deposition. Furthermore, two-dimensional speckle-tracking echocardiography can enhance clinical suspicion of CA and should be recommended as part of the diagnostic workup.

## Introduction

Cardiac amyloidosis (CA), characterized by the deposition of amyloid fibrils in the heart tissue, is a serious and progressive infiltrative disease. The pathognomonic histological feature of CA is the apple-green birefringence observed under polarized light following Congo red staining (1). CA is an underdiagnosed form of restrictive cardiomyopathy, often rapidly progressing to heart failure (HF) (2). While untreated patients face a poor prognosis, effective therapies are now available that can halt disease progression. Early intervention is associated with a better prognosis. These factors underscore the critical importance of a timely diagnosis.

CA has traditionally been considered a rare disorder, posing a significant diagnostic challenge. However, it is now recognized that CA is more common than previously thought, often being underdiagnosed due to its misattribution to more common cardiac diseases or syndromes, particularly in the early stages. Recent advances in imaging techniques and the possibility of achieving a non-invasive diagnosis have significantly improved the detection of CA. Evaluation of CA requires a multimodal approach making use of echocardiography, cardiac magnetic imaging (cMRI), and nuclear imaging (1, 3–6). The diagnosis of CA initially relies on a high level of clinical suspicion, followed by confirmation through a combination of imaging modalities and an endomyocardial or extracardiac biopsy (1).

In general, an increased left ventricle (LV) wall thickness greater than 12 mm, in the absence of other plausible causes of LV hypertrophy, is suggestive of CA. However, the presence of normal wall thickness in a non-dilated LV may indicate a different underlying condition and is easily overlooked. We present a case of a female patient with CA whose diagnosis was initially missed during the echocardiographic examination.

## Case presentation

The 50-year-old female patient presented with worsening breathlessness on exertion, paroxysmal nocturnal dyspnea, oliguria, and lower-extremity edema 6 months prior to being referred to our hospital. She was subsequently admitted to another hospital. Her medical history was unremarkable, with no significant past illnesses, and there was no family history of similar conditions. A chest computed tomography (CT) scan revealed bilateral pleural effusion, pericardial effusion, and pulmonary edema. Ultrasonography indicated congestive liver changes. Selective coronary angiography was normal. The patient’s N-terminal pro-brain natriuretic peptide (NT-proBNP) level was 2,112 pg/ml. Transthoracic echocardiography showed a left ventricular ejection fraction (LVEF) of 51%, with normal LV wall thickness, moderate tricuspid regurgitation, and pulmonary artery systolic pressure of 46 mmHg. Based on these findings, the patient was diagnosed with valvular heart disease and heart failure [New York Heart Association (NYHA) Class IV]. Treatment with spironolactone, furosemide, and β-blockers was initiated to manage systemic congestion. However, the patient discontinued the medications of her own accord after 2 months. Subsequently, edema reappeared in her ankles and progressively involved the entire lower limbs, along with gradually worsening breathlessness.

**Figure 1:**
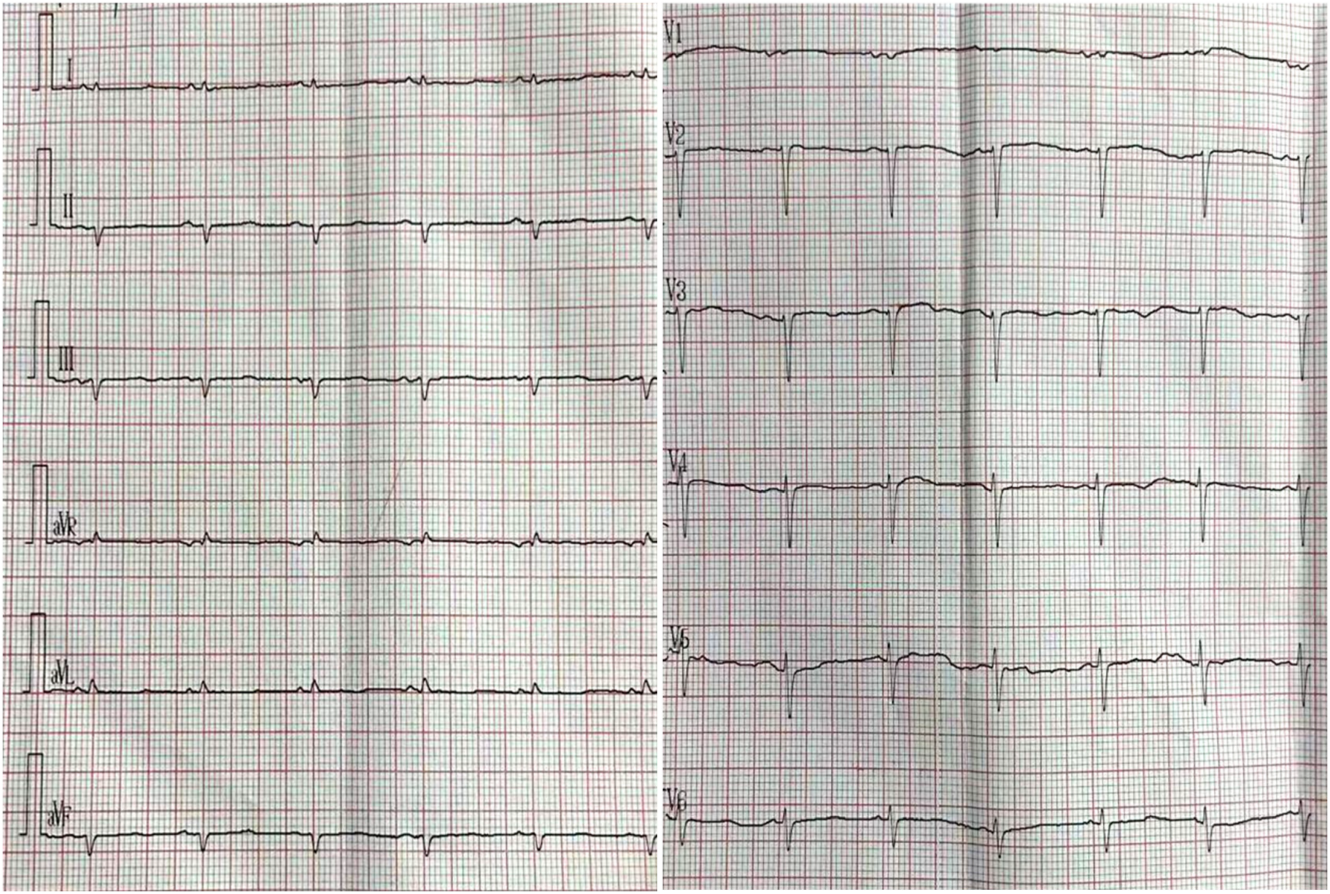
Electrocardiogram. Low QRS voltages (all limb leads <5 mm in height); poor R-wave progression in the chest leads (pseudoinfarction pattern).

**Figure 2:**
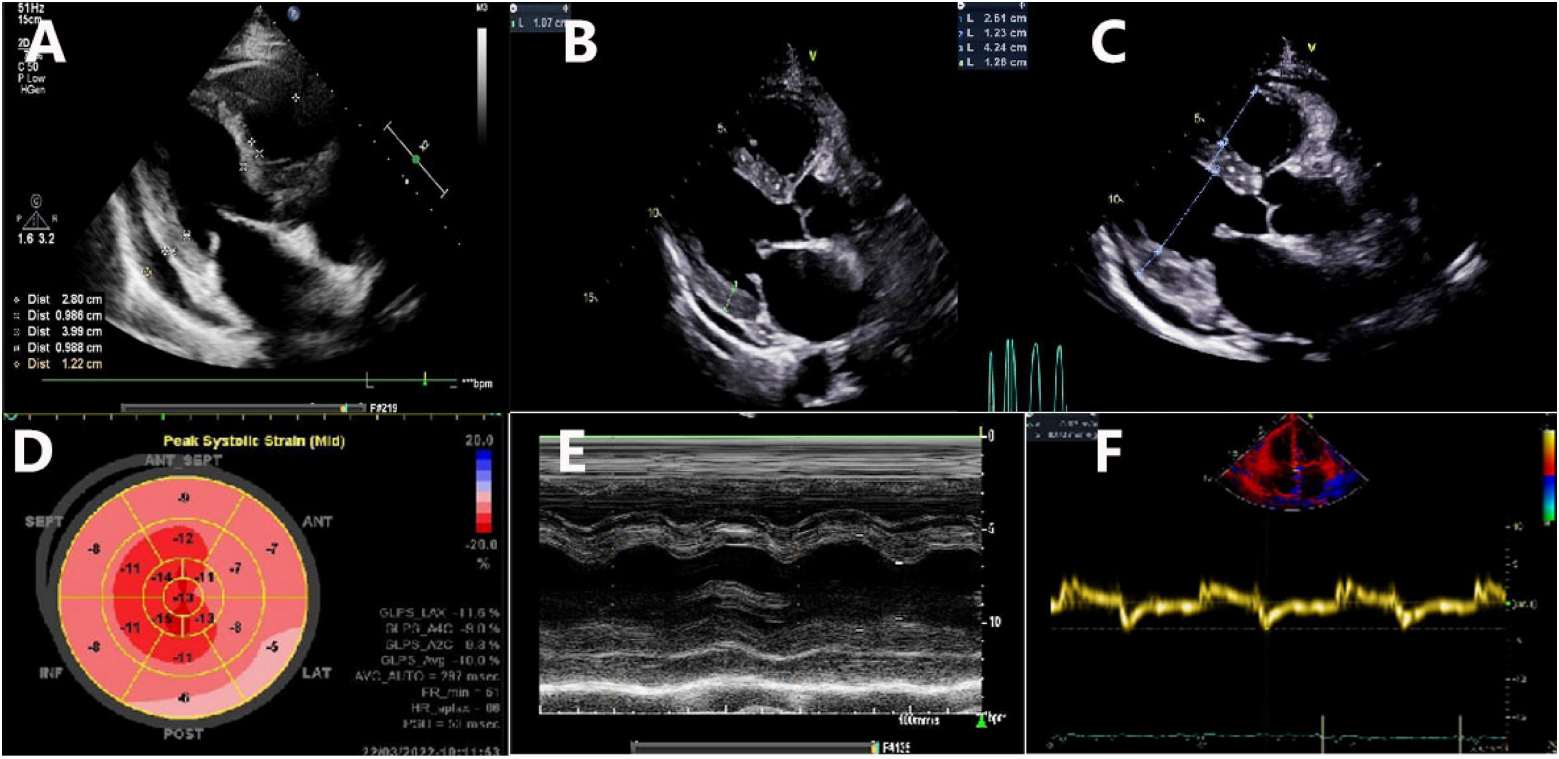
Transthoracic echocardiographic imaging. **(A)** A long axis view shows the normal wall thickness of the IVS (9.86 mm) and LVPW (9.88 mm) obtained on 6 March 2022; **(B)** a long axis view shows the normal wall thickness of the IVS (10.5 mm) and LVPW (10.7 mm) obtained on 22 March 2022; **(C)** a long axis view shows the increased wall thickness of the IVS (12.3 mm) and LVPW (12.6 mm) obtained on 21 May 2022; **(D)** decreased global longitudinal strain (GLS − 10%) and a pattern of relative “apical sparing” of longitudinal strain in the apex of the LV; **(E)** normal left ventricular wall motion; **(F)** reduced tissue Doppler mitral annular velocities at the septal mitral annulus.

The patient was then referred to our outpatient clinic for further evaluation and management. She was 150 cm tall and weighed 60 kg. Her blood pressure was 99/61 mmHg with a pulse rate of 95 beats per minute. A physical examination revealed marked jugular venous distention, moderate hepatomegaly, and bilateral lower-extremity edema. A respiratory examination showed scattered dry and moist rales in both lower lung fields. The laboratory results showed an elevated NT-proBNP level of 3,709 pg/ml (normal <125 pg/ml) and a serum high-sensitivity cardiac troponin I (CTNI) level of 351.28 ng/L (normal <17.5 ng/L) (Table 1). Electrocardiography showed low QRS voltages in the limb leads (all limb leads <5 mm in height) and a pseudoinfarction pattern, characterized by poor R-wave progression in the anterior leads (Figure 1). Echocardiography demonstrated an LVEF of 51% with a restrictive pattern of transmitral flow (medial E/e'15.5, grade 3 diastolic dysfunction) (Figure 2). The left ventricle was non-dilated (LV diameter 40 mm) with normal wall thickness [interventricular septum (IVS) 9.86 mm, left ventricular posterior wall (LVPM) 9.88 mm], and there was no evidence of a granular sparkling echotexture of the myocardium. Mild left atrial (LA) enlargement (39 mm) and mild pericardial effusion were noted. In addition, severe tricuspid regurgitation and moderately elevated pulmonary artery systolic pressure (56 mmHg) were observed (Table 2).

**Table 1:** Timeline.

	Presentation	Investigations	Findings
First hospitalization (September 2021 at another hospital)	Breathlessness on exertion, paroxysmal nocturnal dyspnea, oliguria, and lower-extremity edema	Blood examination	NT-proBNP 2,112 pg/ml
		Echocardiography	LVEF 0.51, moderate tricuspid regurgitation, and pulmonary artery systolic pressure of 46 mmHg
		Chest CT scan	Pleural effusion, pericardial effusion, and pulmonary edema
		Abdominal ultrasonography	Congestive liver
		Coronary angiography	Normal
Second hospitalization (6 March 2022 at our hospital)	Same as above	Blood examination	NT-proBNP 3,709 pg/ml, DDi 1.69 μg/ml, and CTNI 351.28 ng/L
		Electrocardiography	Low voltage in the limb leads and a pseudoinfarction pattern in the anterior leads
		Echocardiography	LVEF 0.52, LA 39 mm, LV 40 mm, IVS 9.86 mm, LVPW 9.88 mm, IVC 22 mm, E/e’15.5, severe tricuspid regurgitation, and pulmonary artery systolic pressure 56 mmHg
Third hospitalization (22 March 2022 at our hospital)	The above symptoms worsened	Blood examination	NT-proBNP 6,152 pg/ml, DDi 1.21 μg/ml, CTNI 674.84 ng/L, an elevated level of free immunoglobulin λ light chains in serum, and a normal concentration of κ-serum-free light chains, with an abnormally low κ/λ ratio
		Serum immunofixation electrophoresis	Precipitation band in the λ lane
		Echocardiography	LVEF 0.44, LA 39 mm, LV 39 mm, IVS 10.5 mm, LVPW 10.7 mm, IVC 19 mm, E/e’ 23.6, GLS − 10%, relative “apical sparing” of LS in the LV apex, moderate tricuspid regurgitation, and pulmonary artery systolic pressure 30 mmHg
		Extracardiac biopsy	Amyloid deposits confirmed by a positive Congo red staining of skin, subcutaneous tissue, muscle, and blood vessel biopsies
Fourth hospitalization (April 2022 at our hospital)	The above symptoms occurred repeatedly	Echocardiography	LVEF 0.46, LA 42 mm, LV 39 mm, IVS 13.1 mm, LVPW 10.8 mm, IVC 22 mm, E/e’ 17.5, GLS − 13.4%, relative “apical sparing” of LS in the LV apex, moderate tricuspid regurgitation, and pulmonary artery systolic pressure 45 mmHg
		Cardiac magnetic resonance imaging	Mild biatrial enlargement; subendocardial delayed gadolinium enhancement of LA, RA, LV, and RV; pleural effusion; and pericardial effusion
		ECT 99m Tc-PYP	CA of ATTR subtypes, pleural effusion, and pericardial effusion
		Bone marrow puncture	Active bone marrow hyperplasia and scattered well-differentiated plasma cells
		Bone marrow immune phenotype	Abnormal plasma cell phenotype: the expression of CD38, CD138, CD56, CD117, CD27^dim, and λ light chains and no expression of CD19, CD45, and κ light chains

NT-proBNP, N-terminal fragment brain natriuretic peptide; LVEF, left ventricular ejection fraction; DDi, D-dimer; CTNI, cardiac troponin; LA, left atrium; LV, left ventricular; IVS, interventricular septum; LVPW, left ventricular posterior wall; IVC, inferior vena cava; GLS, global longitudinal strain; LS, longitudinal strain; RV, right ventricular; ECT 99m Tc-PYP, 99mTechnetium-pyrophosphate scintigraphy; CA, cardiac amyloidosis; ATTR, transthyretin.

**Table 2:** Changes in echocardiographic parameters.

	IVS (mm)	LVPW (mm)	LVEF	GLS	LA (mm)	LV (mm)	RWT	PSBP (mmHg)	E/e’	PE (mm)
6 March 2022	9.86	9.88	0.52	—	39	40	0.49	56	15.5	12
22 March 2022	10.5	10.7	0.44	10.0%	39	39	0.54	30	23.6	8
April 2022	13.1	10.8	0.46	13.4%	42	39	0.61	45	17.5	19
May 2022	12.3	12.6	0.43	—	38	42	0.59	59	19.7	14
August 2022	13.1	11.4	0.47	–9.9%	38	41	0.60	45	15.2	10
March 2023	12.1	11.1	0.22	—	41	32	0.73	30	17.7	11

IVS, interventricular septum; LVPW, left ventricular posterior wall; LVEF, left ventricular ejection fraction; GLS, global longitudinal strain; LA, left atrium; LV, left ventricular; IVC, inferior vena cava; PSBP, pulmonary systolic blood pressure; E, mitral E wave velocity; e’, medial tissue Doppler e’ wave velocity; PE, pericardial effusion.

However, 2 weeks later, the patient's symptoms worsened further, and she was subsequently admitted for further management. Laboratory results revealed NT-proBNP levels of 6,152 pg/ml, D-dimer (DDi) of 1.21 µg/ml, and CTNI of 674.84 ng/L. The κ and λ immunoglobulin light chain (AL) assays showed an elevated level of free λ light chains, with a normal concentration of κ serum-free light chains (FLCs). However, the patient’s κ/λ ratio was abnormally low. Serum immunofixation electrophoresis demonstrated a precipitating band in the λ lane. Repeat quantitative echocardiography showed increased LV wall thickness (IVS 10.5 mm, LVPW 10.7 mm, still within normal range), decreased global longitudinal strain (GLS − 10%), and a pattern of relative “apical sparing” of longitudinal strain in the apex of the LV (Table 2, Figure 2). These findings raised suspicion for a diagnosis of CA.

**Figure 3:**
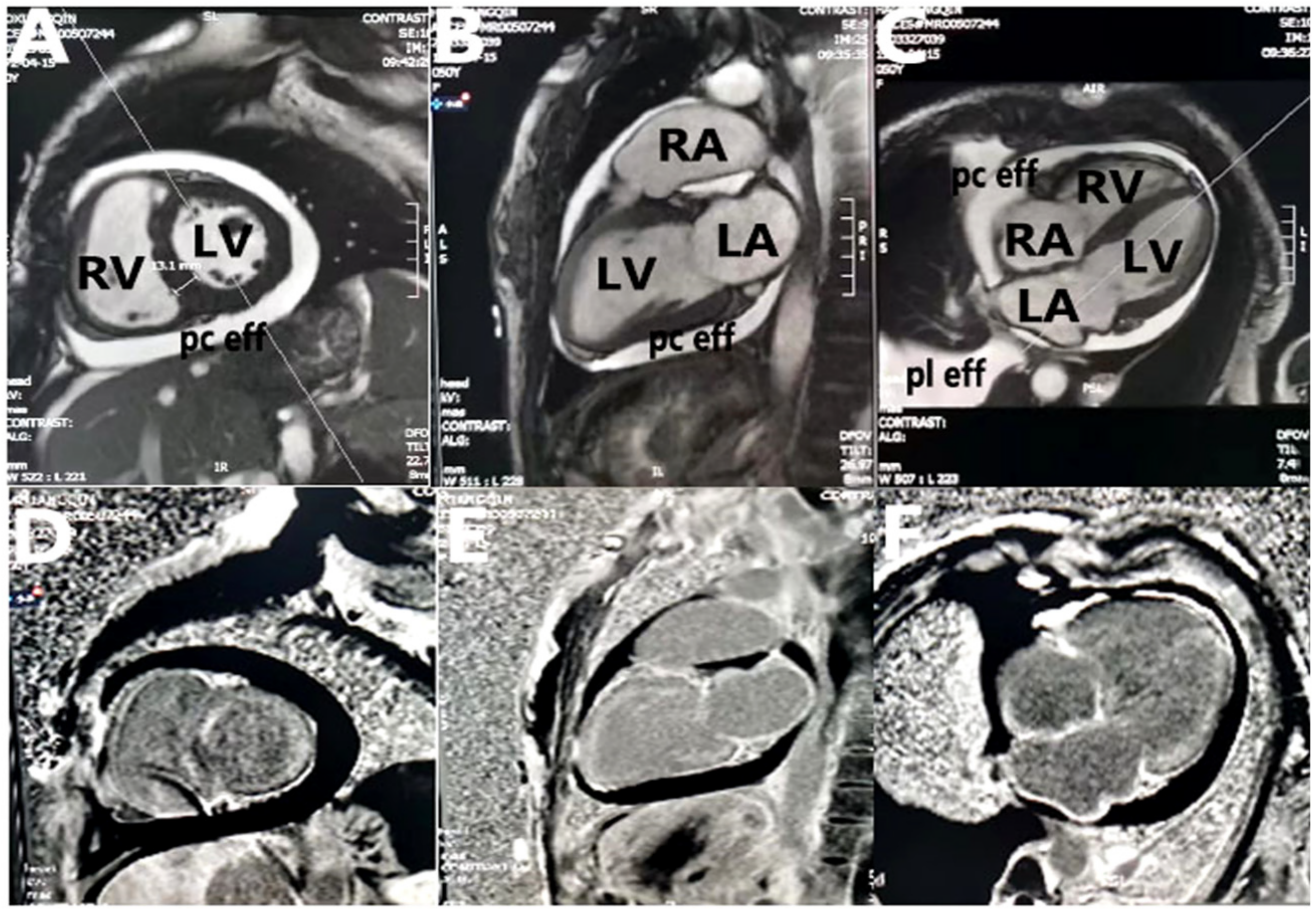
Cardiac magnetic resonance imaging. **(A–C)** Two-chamber, three-chamber, and four-chamber steady-state free precession images demonstrate mild biatrial enlargement, increased left ventricular wall thickness (13 mm), pleural effusion, and pericardial effusion; **(D–F)** two-chamber, three-chamber, and four-chamber views from postgadolinium delayed enhancement images show left atrium, right atrium, right ventricle, and left ventricle line-like enhancement. LA, left atrium; RA, right atrium; LV, left ventricle; RV, right ventricle; pc eff, pericardial effusion; pl eff, pleural effusion.

**Figure 4:**
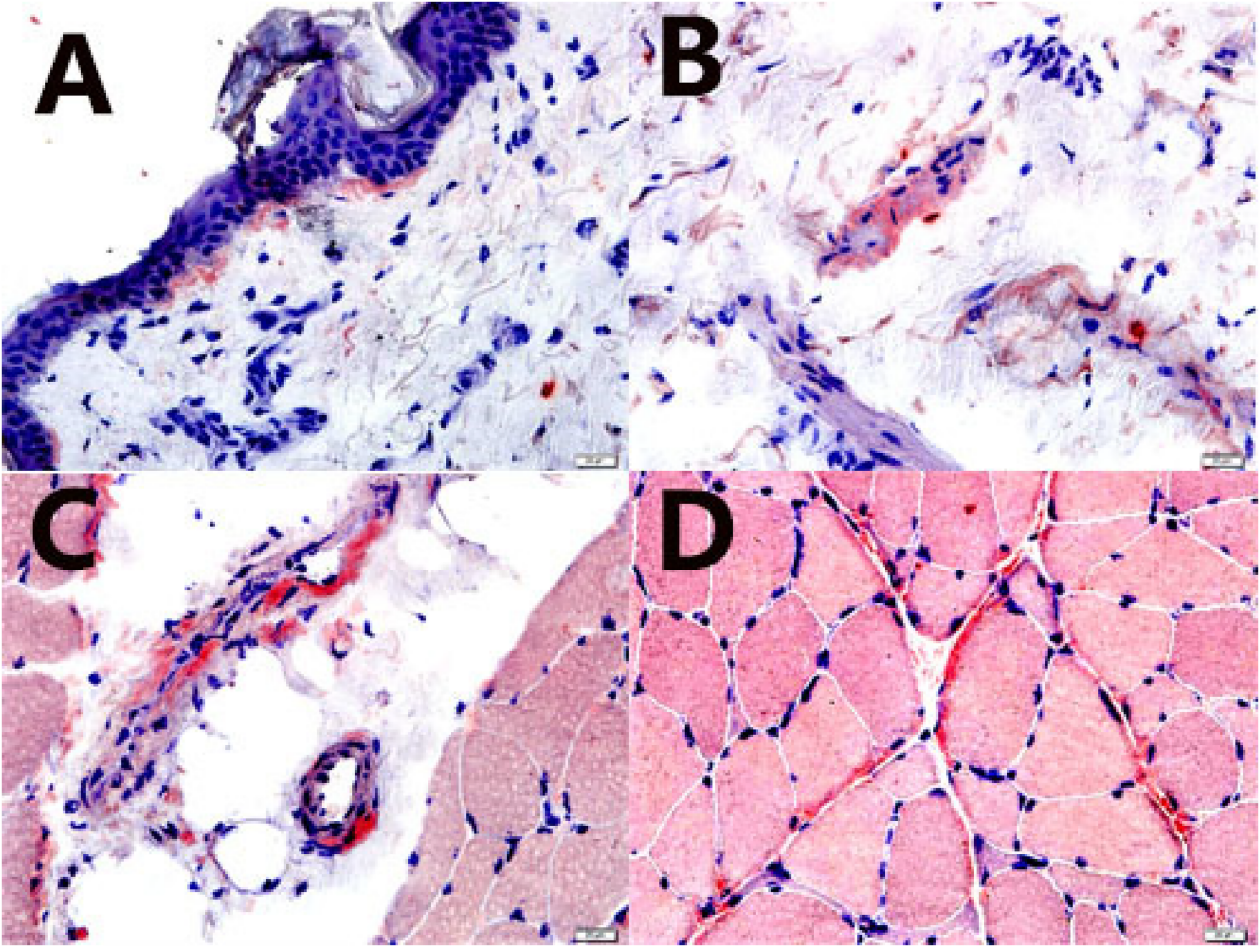
Skin, subcutaneous tissue, blood vessel, and muscle biopsies. Amyloid deposits were confirmed by a positive Congo red stain (characteristic salmon-pink red) (Congo red stain, 400×). **(A)** skin; **(B)** subcutaneous tissue; **(C)** blood vessel; **(D)** muscle (perimysium and endomysium).

**Figure 5:**
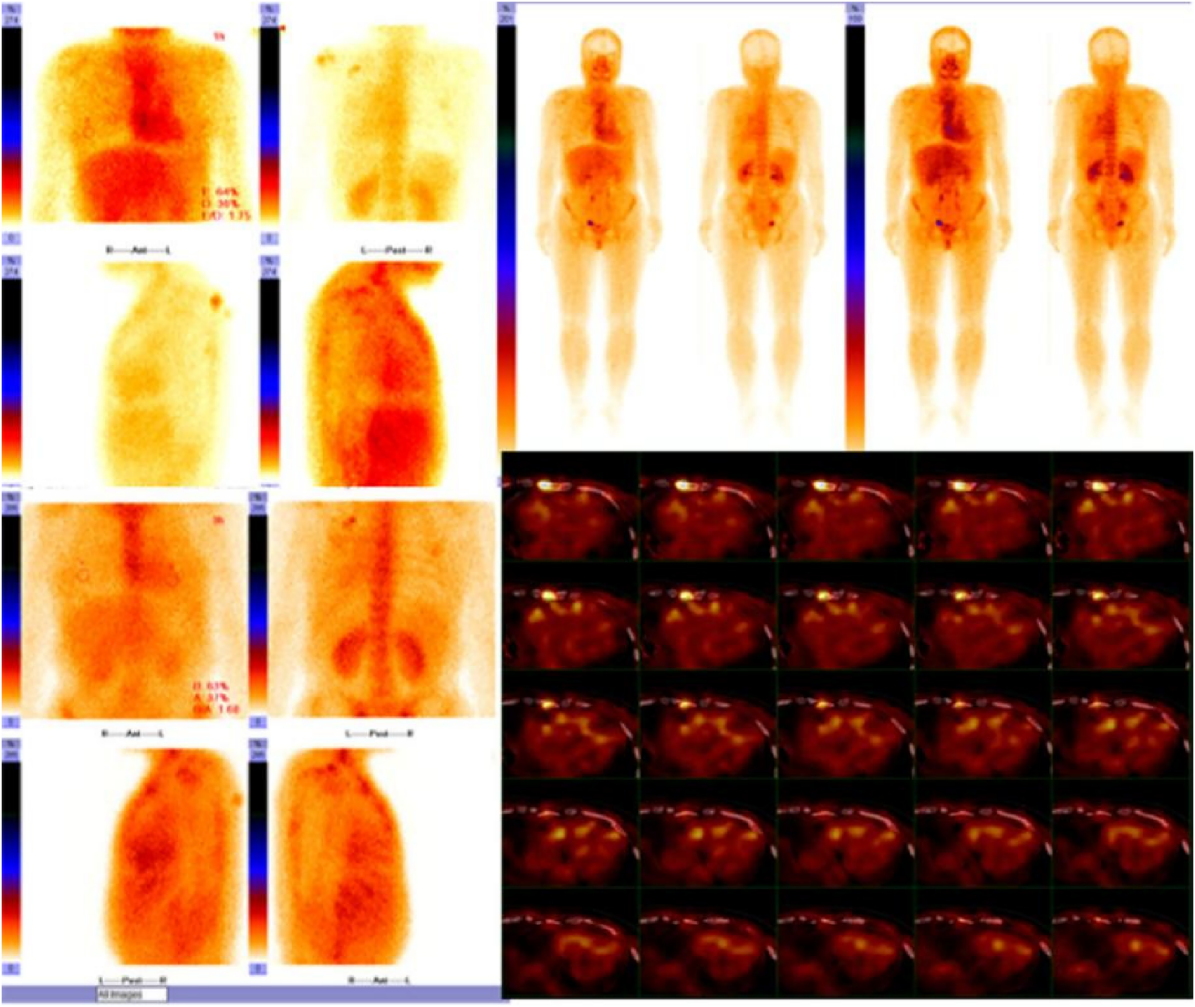
Cardiac uptake in bisphosphonate scintigraphy showing Perugini grade 2 and similar myocardial and bone uptake.

cMRI was performed 1 month later, revealing delayed gadolinium enhancement in the LA, right atrium (RA), LV, and right ventricle (RV), along with pleural and pericardial effusions (Figure 3). Increased LV wall thickness (>12 mm) was confirmed by both cMRI and echocardiography, with the appearance of a granular sparkling echotexture in addition to the aforementioned features. An extracardiac biopsy showed positive Congo red staining for amyloid deposits in the skin, subcutaneous tissue, muscle, and blood vessels (Figure 4). Cardiac uptake in bisphosphonate scintigraphy showed Perugini grade 2 (Figure 5). These findings ultimately led to the diagnosis of CA.

Furthermore, a bone marrow aspiration revealed active bone marrow hyperplasia with scattered well-differentiated plasma cells. The bone marrow immunophenotyping showed positive expression of CD38, CD138, CD56, CD117, CD27^dim^, and λ light chains in the abnormal plasma cell population, while CD19, CD45, and κ light chains were negative.

Based on these findings, the patient was ultimately diagnosed with CA due to light chain (λ ) deposition, accompanied by heart failure. She was treated with furosemide/torasemide, spironolactone, β-blockers, ivabradine, dapagliflozin, bortezomib, daratumumab, and dexamethasone. One month after starting treatment, testing of FLCs revealed a decrease in serum-free λ light chains from 158.27 to 31.70 mg/L (reference range: 5.71–26.30 mg/L), while the free κ/λ ratio increased from 0.1017 to 0.2104 (reference range: 0.26–1.65). Moreover, 3 months post-diagnosis, her NT-proBNP levels decreased from 6,152 to 2,872 pg/ml, reflecting an improvement in her heart failure. Clinically, her heart failure symptoms also showed improvement, and her functional status fluctuated between NYHA Class II and NYHA Class IV. Despite these improvements, the patient continued to experience recurrent episodes of heart failure, often complaining of fatigue and shortness of breath during routine activities, and occasionally even at rest or when lying down. After short-term intensive hospitalization, her symptoms and signs improved temporarily. Unfortunately, the patient ultimately succumbed to heart failure in July 2023.

## Discussion

CA, a form of infiltrative cardiomyopathy, is associated with a poor prognosis in untreated patients. More than 30 distinct types of amyloid precursor proteins have been identified (1, 7). CA is primarily caused by either AL or transthyretin (ATTR) amyloidosis. The interstitial deposition of amyloid fibrils leads to structural changes, including the thickening and stiffening of the cardiac chambers in CA. Consequently, an increased mean LV wall thickness (>12 mm) and free RV wall thickness (>5 mm), in the absence of other plausible causes of ventricular hypertrophy, can be indicative of infiltrative cardiomyopathy (1).

An early diagnosis is crucial for the timely initiation of effective therapies. Despite advancements in diagnostic techniques, diagnosing CA remains challenging and requires a high index of clinical suspicion (1). An endomyocardial biopsy is the gold standard for diagnosing CA, as it demonstrates apple-green birefringence when stained with Congo red and examined under polarized light microscopy. However, due to its associated risks and limited availability, it is not recommended as a first-line diagnostic approach. The diagnosis of CA requires heightened awareness, a high level of clinical suspicion, and expertise in integrating clinical, electrocardiographic, and multimodal imaging data (1, 4).

Echocardiography is the first-line non-invasive cardiac imaging modality (1, 3, 4, 8). It is readily available in most healthcare centers and plays a vital role in the early diagnosis and prognostication of CA (9). According to clinical guidelines, the diagnostic criteria for CA include an echocardiogram showing increased wall thickness and a granular appearance, in combination with a positive non-cardiac biopsy (1). Increased wall thickness in a non-dilated left ventricle should prompt further investigation as normal ventricular wall thickness in CA is extremely rare (10). Left ventricular wall thickening is generally thought to be mandatory for the diagnosis and is part of previous diagnostic criteria. Lee et al. conducted a retrospective analysis comparing patients with varying left ventricular wall thicknesses (10). Nagy et al. conducted a retrospective analysis of cardiac amyloidosis with normal wall thickness and provided valuable information on its clinical characteristics and outcomes (11). These studies provide valuable insight into understanding “thick” vs. “non-thick” presentations of left ventricular wall thickness in CA. Deﬁning cardiac involvement in AL amyloidosis using increased left ventricular wall thickness may result in a signiﬁcant number of undiagnosed cases. In particular, during the early stages, significant left ventricular wall thickening may not yet be evident, but pathological processes are already ongoing. Our case report presents a unique clinical profile of a 50-year-old female patient with CA and normal left ventricular wall thickness, a relatively underexplored patient group. The inclusion of a younger patient with early-stage disease who is symptomatic at this stage adds a novel dimension to the clinical spectrum of AL amyloidosis, an aspect that, to our knowledge, has not been sufficiently addressed in the existing literature.

In general, a definitive diagnosis of CA can only be made after suspicion is raised, and patient outcomes are highly dependent on the timely initiation of therapy. Despite the absence of classical echocardiographic features of amyloid deposition, this case suggests that amyloidosis may be the underlying cause of cardiomyopathy in patients presenting with diastolic dysfunction and pericardial effusion. The normal wall thickness observed could be due to the patient's short stature (150 cm), which may place her at the lower end of the normal range for wall thickness prior to amyloid deposition. Despite significant amyloid infiltration, the wall thickness remained within normal limits at the time of referral to our hospital. The patient became symptomatic early in the disease course before LV wall thickening became pronounced.

Quantitative measurements of wall thickness, chamber dimensions, and cardiac function are crucial, and the normal reference values are essential for distinguishing between normal and abnormal findings. However, variations in wall thickness and cardiac chamber dimensions are largely attributable to differences in body size (12). The use of the current echocardiographic thresholds recommended by guidelines may lead to a significant prevalence of misdiagnosis of abnormal wall thickness, particularly in Chinese patients. Echocardiographic parameters show promising potential for detecting CA. Further studies are needed to establish optimal cut-off values for these echocardiographic variables to improve diagnostic accuracy (9). Relative wall thickness (RWT) is an important parameter. At the patient’s initial echocardiographic examination at our hospital in this case, RWT was measured to be 0.49, which exceeds the normal range, and it gradually increased over time (Table 2). Therefore, it is important to account for the physiological impact of body size on cardiac dimensions by indexing cardiac measurements to body size. In this context, RWT may serve as a more sensitive and reliable indicator. As the infiltrative disease progressed, wall thickness gradually increased. Wall thickness can be considered an index of the burden of myocardial infiltration in CA, but it is not the only marker. Other echocardiographic signs that may suggest the presence of CA include right ventricular wall thickening, prominent biatrial dilation with a normal or small ventricular size, diffuse valve thickening, pericardial effusion in the absence of other plausible causes, and restrictive hemodynamics with relatively preserved ejection fraction (1). In this case, the patient presented with biatrial dilatation, grade 3 diastolic dysfunction, and pericardial effusion.

Given the limitations of standard 2D echocardiography, newer imaging techniques, such as two-dimensional speckle-tracking echocardiography (2D-STE), can be valuable in supporting clinical suspicion during the assessment of CA (13). While advanced diagnostic methods may not always be readily available, they can provide important prognostic information (14). Therefore, 2D-STE should be considered as a recommended approach. It is often more sensitive and can help differentiate CA from other causes of cardiac hypertrophy. In this case, reduced GLS with apical sparing was observed.

However, an echocardiogram alone cannot confirm the diagnosis. To confirm the diagnosis of suspected CA, it is essential to integrate findings from clinical representations, an electrocardiogram (ECG), an echocardiogram, cMRI, and an endomyocardial and/or non-cardiac biopsy. In addition, an ECG can provide further assessment of amyloid (15). An ECG showing low voltage along with concentrically increased wall thickness is highly suggestive of CA. In our case, low QRS voltages (all limb leads <5 mm in height) with poor R-wave progression in the chest leads (a pseudoinfarction pattern) were observed before any increase in LV wall thickness was demonstrated. cMRI should be considered a non-invasive option for screening CA. Due to the extracellular deposition of amyloids, CA is characterized by global subendocardial late gadolinium enhancement (4, 16).

Given the complexity of the clinical scenario, no single diagnostic test is definitive. Instead, different techniques are valuable at various stages of the diagnostic workup. In our case, positive findings from blood tests, an electrocardiogram, an echocardiogram, cMRI, and a non-cardiac biopsy ultimately confirmed the diagnosis of light-chain (λ) CA. It seems reasonable to screen patients with non-increased left ventricular wall thickness heart failure with preserved ejection fraction (HFpEF) for CA, especially if other causes of HF and elevated BNP levels (severe renal failure, fluid retention, severe anemia, clinically significant bradycardia or tachycardia, or arterial-venous shunts) can be excluded (10, 11). Deﬁning cardiac involvement in AL amyloidosis using increased left ventricular wall thickness (LVWT) may result in a signiﬁcant number of undiagnosed cases, and more sensitive methods, such as cardiac MRI or a myocardial biopsy, for the early detection of amyloid inﬁltration of the heart are needed. Because complete hematological remission, age, E/e’, and NT-proBNP level are independent predictors of survival in patients with AL amyloidosis, an early diagnosis and appropriate chemotherapy may improve survival rates in patients with AL amyloidosis (10). In this case report, we present a case of CA without increased wall thickness and without the characteristic granular sparkling echotexture in a non-dilated LV. However, the early clinical and biomarker changes were already evident, including elevated NT-proBNP and high-sensitivity troponin levels. This case highlights the dynamic progression of CA and underscores the importance of early detection and intervention.

## Conclusion

CA can often be overlooked in patients with normal LV wall thickness and a non-dilated LV as these patients could be in the early stages of the disease. In smaller individuals, RWT may serve as a more useful parameter to prompt further investigation. For patients presenting with increased RWT, restrictive filling, and pericardial effusion in the absence of other likely causes, CA should be considered, even in the absence of the classical echocardiographic features of amyloid deposition. Two-dimensional speckle-tracking echocardiography can be a valuable tool to support clinical suspicion and should be recommended as part of the diagnostic workup.

## Data Availability

The original contributions presented in the study are included in the article/Supplementary Material, further inquiries can be directed to the corresponding author.
